# Lifestyle intervention to reduce cardiovascular risk in kidney transplant recipients: the KT-LIFESTYLE multicentre randomized controlled trial

**DOI:** 10.1093/ckj/sfag153

**Published:** 2026-05-12

**Authors:** Valentina Totti, Gaia Tabanelli, Michele Stecchi, Lucia Brodosi, Valeria Grandinetti, Valeria Pizzuti, Paola Emanuela D’Intino, Daniele Ferrarini, Eleonora Capelli, Rocco Di Michele, Giovanni Mosconi, Vittorio Albertazzi, Erika Cordella, Patrizia Brigidi, Maria Cristina Morelli, Gaetano La Manna, Giuseppe Tarantino

**Affiliations:** Internal Medicine Unit for the Treatment of Severe Organ Failure, IRCCS Azienda Ospedaliero – Universitariadi Bologna, Italy; Clinical Nutrition and Metabolic Division, IRCCS AOUBO, Bologna, Italy; Clinical Nutrition and Metabolic Division, IRCCS AOUBO, Bologna, Italy; Clinical Nutrition and Metabolic Division, IRCCS AOUBO, Bologna, Italy; Nephrology, Dialysis and Kidney Transplant Unit, IRCCS Azienda Ospedaliero – Universitariadi Bologna, Bologna, Italy; Nephrology, Dialysis and Kidney Transplant Unit, IRCCS Azienda Ospedaliero – Universitariadi Bologna, Bologna, Italy; Sports Medicine Unit, AUSLBologna, Italy; Sports Medicine Unit, AUSLBologna, Italy; Clinical Nutrition and Metabolic Division, IRCCS AOUBO, Bologna, Italy; Department for Life Quality Studies, University of Bologna, Bologna, Italy; GdS“Esercizio fisico nel paziente con insufficienza renale cronica” of the Società Italianadi Nefrologia, Forlì, Italy; Nephrology and Dialysis Unit, Forlì-Cesena, AUSL Romagna, Italy; Coordination for Transplants Center (CRT-ER), IRCCS Azienda Ospedaliero – Universitariadi Bologna, Italy; Human Microbiomics Unit, Department of Medical and Surgical Sciences, University of Bologna, Italy; Internal Medicine Unit for the Treatment of Severe Organ Failure, IRCCS Azienda Ospedaliero – Universitariadi Bologna, Italy; Nephrology, Dialysis and Kidney Transplant Unit, IRCCS Azienda Ospedaliero – Universitariadi Bologna, Bologna, Italy; Coordination for Transplants Center (CRT-ER), IRCCS Azienda Ospedaliero – Universitariadi Bologna, Italy

**Keywords:** cardiovascular risk, dietary counselling, exercise, kidney transplantation, lifestyle intervention

## Abstract

**Background:**

Kidney transplant recipients remain at high cardiovascular risk despite improved graft and patient survival. Physical inactivity, weight gain, and suboptimal dietary habits are common after transplantation and may contribute to this burden.

**Methods:**

The KT-LIFESTYLE trial is a pragmatic, multicentre, prospective, open-label randomized controlled study designed to evaluate whether a structured lifestyle intervention can reduce cardiovascular risk in kidney transplant recipients. Participants will be randomized 1:1 to individualized exercise prescription plus tailored dietary counselling or standard lifestyle advice. The intervention combines multidisciplinary assessment, individualized exercise programming, motivational interviewing, and nutritional counselling integrated into routine transplant follow-up. The primary endpoint is the change in 10-year cardiovascular risk, assessed by the Framingham score over 36 months. Secondary outcomes include renal function estimated by Chronic Kidney Disease Epidemiology Collaboration (CKD-EPI) 2021 equation estimated glomerular filtration rate, body composition, inflammatory markers, gut microbiota composition, health-related quality of life, adherence to physical activity and dietary counselling, hospital admissions, major adverse cardiovascular events, and all-cause mortality.

**Conclusions:**

KT-LIFESTYLE aims to provide evidence on the effectiveness and feasibility of a long-term lifestyle intervention after kidney transplantation. The study may also clarify the mechanisms through which lifestyle modification influences cardiovascular risk, inflammation, body composition, and gut microbiota in this population.

Clinical trials registration number: NCT06806670

## Graphical Abstract

**Graphical Abstract:**
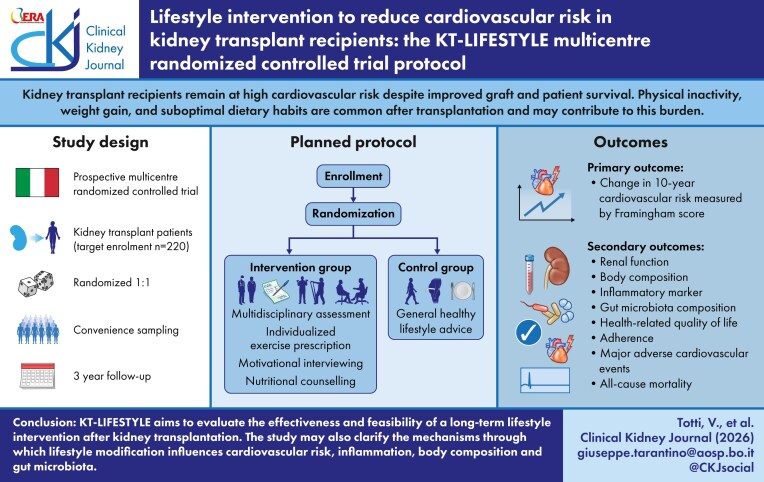

KEY LEARNING POINTS **What was known:**

- Both physical inactivity and unhealthy diet, known modifiable cardiovascular risk factors, are highly prevalent after kidney transplantation.
- Exercise training is safe and beneficial after kidney transplantation.
- Integrating exercise and tailored diet as part of daily life after kidney transplantation is highly recommended.

**This study adds:**

- KT-LIFESTYLE will evaluate whether combined physical exercise and dietary counselling reduce cardiovascular risk in kidney transplant recipients.
- The study will provide evidence on the efficacy and adherence to ‘exercise and diet therapy’ correlated with lifestyle modification.
- Implementation outcomes will provide insights on successful strategies to promote sustained lifestyle intervention in this population.

**Potential impact:**

- Results may contribute to guidelines on lifestyle interventions after kidney transplantation.
- Findings will inform the development of implementation strategies to reduce cardiovascular risk after kidney transplantation and to promote sustained lifestyle changes for improved long-term outcomes.
- This study could help clarify the impact of lifestyle on the gut microbiota and elucidate the relationship between the relative abundance of different bacterial taxa and cardiovascular risk.

## INTRODUCTION

Solid organ transplantation substantially improves survival and quality of life in patients with end-stage organ disease, allowing recovery of physical function and social and work reintegration. Despite these advances, kidney transplant recipients (KTRs) remain at markedly increased risk of cardiovascular disease, a leading cause of late morbidity and mortality in this population [1]. Observational studies consistently show that cardiovascular events occur more frequently in KTRs than in nontransplanted individuals, particularly in the presence of diabetes or established vascular disease [2]. Among modifiable risk factors, physical inactivity and unhealthy dietary habits are common after kidney transplantation and contribute to weight gain, metabolic complications, and worse graft and patient outcomes [3]. Current international recommendations, including World Health Organization (WHO) guidelines, advocate regular physical activity and healthy eating patterns for people with chronic diseases and transplant recipients [4]. Randomized and longitudinal studies in KTRs have shown that structured exercise programs, with or without dietary counselling, can improve aerobic capacity, body composition, metabolic profile, and health-related quality of life, although most trials are small, short-term, and rarely embedded in routine clinical pathways [3, 5]. Furthermore, longitudinal studies indicate that maintaining an active lifestyle is associated with better preservation of renal function over time, and that regular weekly training may counteract cardiovascular risk and slow renal function decline in kidney transplant patients [6]. In routine clinical practice, lifestyle counselling remains unstructured, heterogeneous across centres, and insufficiently standardized. Cardiovascular risk prediction tools may also help identify KTRs at higher risk of cardiovascular events. Several studies have applied the Framingham score in patients with chronic kidney disease (CKD) to predict cardiovascular events, although it may underestimate absolute risk in this population [7, 8]. Other studies suggest that, when combined with estimated glomerular filtration rate (eGFR), the Framingham score may help identify KTRs at increased risk of cardiovascular events and post-transplant renal dysfunction [9, 10]. In this context, the KT-LIFESTYLE trial aims to evaluate the effectiveness of a structured lifestyle intervention combining individualized exercise prescription and tailored dietary counselling in KTRs. We hypothesize that a 3-year intervention will reduce cardiovascular risk, assessed using the Framingham score, compared with standard care. The integrated modification of physical activity and dietary habits may therefore play a key role in cardiovascular risk reduction, and the novelty of this study lies in evaluating the potential of an integrated lifestyle intervention in reducing cardiovascular risk in KTRs.

## MATERIALS AND METHODS

### Study design and population

The KT-LIFESTYLE study is a pragmatic, multicentre, prospective, open-label, nonpharmacological randomized controlled trial. The primary endpoint is the between-group difference in change in the Framingham score over 36 months in KTRs receiving the lifestyle intervention versus standard of care. The Framingham score estimates the 10-year probability of a cardiovascular event using routinely collected clinical variables including sex, age, systolic blood pressure, smoking status, diabetes, high-density lipoprotein (HDL) cholesterol, and total cholesterol [11]. Secondary endpoints include renal function progression, assessed by eGFR calculated with the Chronic Kidney Disease Epidemiology Collaboration (CKD-EPI) 2021 formula [12], gut microbiota composition, body mass index (BMI), inflammatory markers [serum C-reactive protein (CRP), interleukin-6 (IL-6), and ferritin], metabolic assessment variation (including glucose and lipid profile) and adherence to physical activity counselling assessed by the International Physical Activity Questionnaire (IPAQ) [13], adherence to dietary counselling assessed by the Food Frequency Questionnaire (FFQ) [14], quality of life assessed by the Short Form-12 Health Survey (SF-12) [15], hospital admissions for significant clinical events, fatal and nonfatal cardiovascular events (major adverse cardiovascular events), and all-cause mortality. A maximal cardiopulmonary exercise test will be performed in a subgroup of 25 participants per group. No comparisons with the general population or other patient groups are planned. Annual interim analyses will assess data completeness, quality, and study conduct. Participants will be recruited from IRCCS AOU Bologna and AUSL Romagna Hospitals. The study population will include clinically, surgically, and functionally stable adult KTRs of both sexes followed in the outpatient setting. Participant enrolment will be based on the inclusion and exclusion criteria presented in Table 1. Written informed consent will be obtained from all participants, who will also complete baseline questionnaires on smoking status, alcohol consumption, pretransplant dialysis vintage, time since transplantation, cause of kidney failure, and comorbidities. Ethical approval (EM221-2025_138/2023/Sper/AOUBo) was obtained from the ethics committee. The study complies with the International Conference on Harmonization Good Clinical Practice guidelines and the Declaration of Helsinki.

### Randomization

Participants will be randomized 1:1 to the lifestyle intervention group, receiving individualized exercise prescription plus dietary counselling, or to the control group, receiving standard lifestyle advice. Randomization will be stratified by sex and age in 5-year intervals and performed using a secure electronic randomization system [16].

### Study interventions

**Figure 1:**
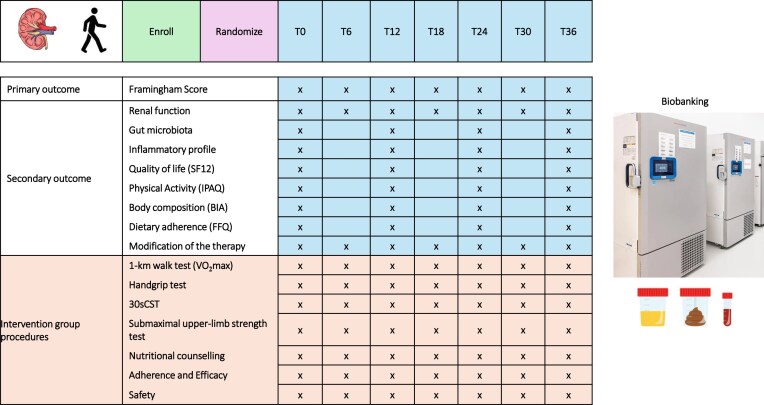
Schematic overview of the participant timeline and study outcomes measured per timepoint. T: timepoints (months); VO_2_max: maximum oxygen uptake; 30sCST: 30-s chair stand test.

Outcome parameters will be assessed at predefined timepoints during routine clinical follow-up (Fig. 1).

In both groups, visits will be aligned with standard transplant care and limited to clinically stable patients without acute cardiological or surgical conditions. Routine evaluation includes blood tests, standard 12-lead electrocardiogram (ECG), cardiological assessment, and spirometry.

All enrolled participants will complete the SF-12 and IPAQ. Nutritional status will be assessed by anthropometry, bioelectrical impedance analysis (BIA), and dietary habits will be evaluated by the FFQ. Blood, urine, and faecal samples will be collected in the fasting state and stored for biobanking.

No changes to immunosuppressive therapy are planned; any adjustments will be made only by the transplant team according to routine practice.

### Lifestyle intervention

Once randomized, participants in the lifestyle intervention group will be referred to the sports medicine unit and then to the dietitian.

The physical activity intervention, through a multidisciplinary team composed of a sports physician and an exercise physiologist, will prescribe an individualized exercise program based on health status and physical fitness assessments [17–19]. Aerobic capacity will be assessed with a 1-km treadmill test for indirect estimation of VO₂max [20], while muscle strength will be measured by handgrip dynamometry [21], the 30-s sit-to-stand test [22], and submaximal strength tests of the upper-limb muscle groups using free weights [23, 24]. Exercise prescription will follow the 2020 WHO guidelines for individuals with chronic diseases, after excluding contraindications to exercise [25]. Adherence and progression will be monitored through scheduled telephone contacts every 2 months in the first year and every 3 months thereafter, using a motivational interviewing approach [26].

**Figure 2:**
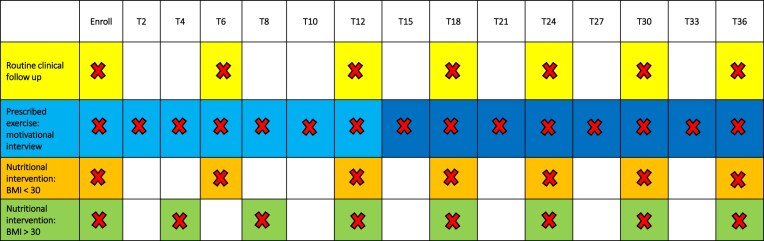
Contact frequency and type throughout the 36-month lifestyle intervention. T: timepoints (months).

The nutritional intervention will promote healthy dietary habits to reduce cardiovascular risk; participants with a BMI <30 will receive nutritional counselling every 6 months, whereas those with a BMI ≥30 will follow an individualized weight-loss diet with reinforcement visits every 4 months in the first year and every 6 months thereafter [27] (Fig. 2).

### Standard of care

Participants in the control group will receive general advice on physical activity and healthy eating, without individualized prescriptions from the transplant centre, reinforced during routine follow-up visits.

### Adherence and efficacy

Adherence to physical activity counselling will be assessed with IPAQ [13, 28], and adherence to dietary counselling with FFQ [29, 30]. Efficacy will be evaluated through routine clinical assessments, including blood chemistry parameters related to immunosuppressive therapy and cardiovascular risk, as well as nutritional status and dietary habits evaluation.

### Study outcomes

Outcome parameters will be assessed at predefined timepoints, as shown in Fig. 1. Study procedures will be performed in the specialized laboratories of IRCCS AOU Bologna and AUSL Romagna Hospitals according to standardized operating procedures. The sequence of assessments will be consistent across centres and timepoints.

### Primary outcome

The primary outcome is the change in estimated 10-year cardiovascular risk over 36 months, measured by the Framingham cardiovascular risk score. The primary analysis will compare the change in Framingham score between the lifestyle intervention and standard-of-care groups. The study is powered to detect a clinically meaningful between-group difference of 1 percentage point in the Framingham score.

### Secondary outcomes

#### Renal function

Renal function will be assessed by comparing eGFR, 24-h proteinuria, and urine albumin-to-creatinine ratio between the two groups. These measurements are collected according to clinical practice at each outpatient visit.

#### Gut microbiota

Gut microbiota composition will be analysed in faecal samples by 16S ribosomal RNA (rRNA) gene amplicon sequencing. After standardized collection, samples will be stored at low temperature, DNA will be extracted using validated kits, and the bacterial 16S rRNA gene will be amplified and sequenced on a high-throughput platform. Bioinformatic processing will provide relative abundances of the main bacterial taxa, which will be compared between groups.

#### Inflammatory profile

The inflammatory profile will be evaluated on fasting venous blood samples collected under standardized conditions. CRP and ferritin will be measured by high-sensitivity immunoassays, and IL-6 by multiplex immunoassay platforms. Samples will be processed promptly and stored at approximately −80°C until batch analysis.

#### IRCCS AOUBO research biobank

Additional blood, urine, and faecal samples will be collected and processed according to standardized biobanking procedures to ensure long-term sample integrity. Blood will be collected in appropriate tubes, and serum and plasma will be aliquoted and stored at −80°C. Urine will be collected as spot samples, aliquoted after laboratory processing, and stored at −80°C. Faecal samples will be self-collected, aliquoted, and rapidly frozen at −80°C to preserve microbial DNA and metabolites. All samples and clinical data will be pseudonymized and managed within the IRCCS biobank infrastructure in compliance with national and European standards.

#### IPAQ—International Physical Activity Questionnaire

The IPAQ is a self-report questionnaire used to assess physical activity in adults. The short form measures walking, moderate- and vigorous-intensity activity, and sitting time over the previous 7 days; responses are converted into metabolic equivalents of task (MET) -minutes per week and classified as low, moderate, or high physical activity levels using standardized scoring protocols [13].

#### Dietary adherence

Dietary intake will be assessed using a validated semiquantitative FFQ previously developed for Italian adults [14]. It captures habitual intake over the preceding 12 months by recording the frequency and portion size of foods and beverages, and nutrient intake will be estimated using standardized food composition databases.

#### Body composition

Body composition including fat-free mass, fat mass, and derived indices such as the fat-free mass index (FFMI) will be assessed by standardized multifrequency BIA according to European Society for Clinical Nutrition and Metabolism (ESPEN) recommendations [31].

### Intervention group procedures

In the intervention group, sports medicine centres will perform validated functional assessments, including the following tests.

#### 1-km walk test

The 1-km walk test is a submaximal test performed on a level treadmill at a constant, moderate intensity, monitored with the Borg 6–20 scale. When perceived exertion reaches 11–13, the test begins, and heart rate, walking speed, completion time, and perceived exertion are recorded to estimate aerobic capacity (VO₂peak/VO₂max) using validated equations [20].

#### Handgrip strength test

Handgrip strength is measured with a handheld dynamometer. Participants are usually seated with the shoulder adducted, elbow at about 90 degrees, and forearm in neutral position. They perform three maximal trials per hand, and the highest value is used for analysis [32].

#### 30-s sit-to-stand test

The 30-s sit-to-stand test is performed on a standard chair without armrests. Participants sit with feet flat on the floor and arms crossed over the chest, then stand and sit as many times as possible in 30 s. The score is the number of correctly completed stands [22].

#### Submaximal upper-limb strength test with free weights

Submaximal testing with free weights involves selecting an exercise for a specific upper-limb muscle group, such as biceps curl, triceps extension, or shoulder abduction, and choosing a load that can be lifted for 8–12 repetitions without volitional fatigue. After a standardized warm-up, repetitions are performed until full movement can no longer be completed, and the result is used to estimate maximal strength through prediction equations [24].

The nutritional intervention will be performed according to individual needs. BMI, eGFR, body composition, total estimated energy expenditure, caloric intake, personal food choices, and palatability will be taken into consideration to promote adherence to Italian nutritional guidelines to reduce cardiovascular risk considering simple sugars, lipid, and fibres intake. Protein intake will be tailored to each individual needs based on eGFR, age, body composition, BMI, and physical activity levels.

In those with a BMI >30, a personalized diet will be redacted aimed to also to obtain significant weight loss while simultaneously reducing cardiovascular risk.

### Statistical considerations

#### Sample size calculation

The sample size was estimated a priori based on the primary endpoint, the Framingham score. The study was powered to detect a between-group difference of 1 point, using parameters from a previous study with similar characteristics [33], including a standard of deviation (SD) of 8.1 and a correlation between repeated measurements of 0.95. With α = 0.05 and power of 80%, 198 participants completing follow-up (99 per group) were required. Assuming a 10% dropout rate, 220 participants will be enrolled; considering an expected 80% participation rate among eligible patients, ∼275 patients will be screened.

#### Statistical analysis plan

All analyses will follow the intention-to-treat principle. Baseline characteristics will be summarized using mean ± SD or median (interquartile range) for continuous variables and counts and percentages for categorical variables. The distribution of variables will be examined before inferential analyses. The primary endpoint will be analysed using repeated-measures analysis of covariance (ANCOVA) , with group, time, and group-by-time interaction as fixed effects and age, time from transplantation, and dialysis duration as covariates. The same framework will be applied to secondary continuous outcomes, including eGFR, gut microbiota composition, BMI, inflammatory markers, and quality of life. Potential effect modifiers will include sex, pretransplant and current physical activity, and dietary characteristics. Mean between-group differences in change in Framingham score will be reported with 95% confidence intervals, and subgroup analyses will be performed by sex and age group. If model assumptions are not met, nonparametric analyses will be used. Discrete outcomes, including hospital admissions, cardiovascular events, and death, will be analysed using binomial or multinomial logistic regression. Safety will be summarized by the frequency and type of adverse events in each group. Data from all centres will be pooled for analysis.

**Table 1:** Inclusion and exclusion criteria.

Male and female patients who are KTRs stabilized from a clinical standpoint (judgment by the reference transplant centre) Minimum age: 30 years completed Maximum age: 69 years completed Time from transplant: from 6 months (after achieving clinical stability) to 10 years Organ function: eGFR (CKD-EPI 2021 formula) ≥30 ml/min/1.73 m² Obtaining informed consent
Patients unable to follow the prescription Recent acute cardiovascular event (<2 months) Unstable angina Hyperkinetic/hypokinetic arrhythmias not controlled with therapy, and with signs of haemodynamic compromise Severe aortic stenosis Heart failure class NYHA III and IV, ejection fraction (EF) <40% Acute diseases limiting physical activity practice Severe hypertension (baseline blood pressure (BP) ≥200/110 mmHg) Neuro-musculoskeletal pathologies that may be aggravated by exercise Patients unwilling to change lifestyle Any form of substance abuse, psychiatric disorder, or condition that, according to the investigator, may complicate physician–patient communication Pregnant women

## DISCUSSION

The KT-LIFESTYLE trial addresses an important unmet need in KTRs, who remain at high cardiovascular risk despite improved graft survival and advances in immunosuppressive therapy. Physical inactivity, weight gain, and inadequate dietary habits are common after transplantation and likely contribute to this risk, particularly in patients with diabetes or established vascular disease [34]. Although randomized and longitudinal studies suggest that exercise and dietary interventions can improve aerobic capacity, body composition, metabolic profile, and quality of life, most have been small, short-term, and not designed to assess global cardiovascular risk as a primary outcome [35, 36]. A key strength of this trial is the use of the 10-year Framingham cardiovascular risk score as an integrated endpoint. Although the Framingham model may underestimate absolute risk in CKD populations, its combination with eGFR has shown reasonable performance as a marker of relative risk and may represent a meaningful intervention target [8, 37]. To our knowledge, KT-LIFESTYLE is among the first multicentre randomized studies in KTRs powered to detect a clinically meaningful reduction in 10-year cardiovascular risk over 3 years through a structured lifestyle intervention. Another strength is the integration of clinical, mechanistic, and patient-centred outcomes. Anthropometric measurements and body composition evaluated using BIA, including FFMI, may better characterize each individual more precisely than BMI alone. In addition, gut microbiota profiling and inflammatory markers such as CRP, IL-6, and ferritin may help clarify biological pathways linking lifestyle modification to cardiovascular and renal outcomes. The use of validated instruments such as the IPAQ, SF-12, and FFQ further strengthens the assessment of behavioural and patient-reported outcomes.

Methodologically, the trial is pragmatic and multicentre, with concealed randomization through REDCap and stratification by age and sex. Individual randomization was deliberately chosen over cluster randomization primarily because the risk of cross-contamination in KT-LIFESTYLE is structurally limited. Furthermore, the ACT trial—the most directly comparable multicentre randomized controlled trial of exercise and diet in KTRs [3]—also employed individual randomization in a multicentre open-label design without reporting significant contamination issues. Finally, cluster randomization would have required a substantially larger sample size to account for the design effect, which was not feasible given the available patient population.

The embedding in routine follow-up, together with individualized exercise prescription, nutritional counselling, motivational interviewing, and scheduled contacts, enhances real-world relevance and may inform future guideline development. The following limitations should also be acknowledged. Open-label design may introduce performance and detection bias, particularly for behavioural and self-reported outcomes, although the primary endpoint is based on objective clinical variables.

In addition, adherence to exercise and diet is self-reported through the IPAQ and FFQ, which may be affected by recall and social desirability bias in the absence of objective measures such as accelerometry or dietary biomarkers. Concerning scalability, the study intervention is resource-intensive, requiring close coordination among nephrology, sports medicine, and nutrition teams, which may be a challenge in other settings.

KT-LIFESTYLE aims to address a major gap in the long-term management of KTRs by evaluating the efficacy in reducing cardiovascular risk through a lifestyle intervention based on physical activity and nutritional counselling.

This study also aims to evaluate feasibility, implementation with routine clinical practice, and scalability.

## Data Availability

No new data were generated in support of this research protocol.
